# A complex presentation of an uncommon disease: Gas-forming pyogenic liver abscess complicated by septic pulmonary emboli and muscle abscesses, a case report and review of the literature

**DOI:** 10.1016/j.idcr.2022.e01673

**Published:** 2022-12-22

**Authors:** Aseel H. Alzibdeh, Ans A. Alamami, Mohammad Adam, Muna Almaslamani, Hamad Abdel Hadi

**Affiliations:** a Communicable Diseases Centre, Hamad Medical Corporation, Qatar; b Department of Critical Care, Hamad General Hospital, Hamad Medical Corporation, Qatar; c Weill Cornell Medicine Qatar, Qatar

**Keywords:** AST, aspartate transaminase, ALT, alanine aminotransferase, HbA1c, glycated hemoglobin, CTPA, computed tomography pulmonary angiogram, HIV, human immunodeficiency virus, GFPLA, Gas-forming pyogenic liver abscess, PLA, Pyogenic liver abscess, Liver abscess, Klebsiella, Septic emboli, Gas forming, DM

## Abstract

**Background:**

Pyogenic liver abscess (PLA) is the most common type of visceral abscess. Its variable clinical presentation depends on patient demography, underlying conditions, causative pathogens as well as the size of the abscess. Most cases are secondary to enteric pathogens that cause focal liver disease. Gas-forming pyogenic liver abscess (GFPLA) is a rare subgroup of PLA characterized by the presence of gas within the abscess. The disease is associated with diabetes mellitus (DM) while Klebsiella *penumoniae* is the most frequently isolated pathogen. Despite appropriate evaluation and management, secondary complications are common with significant morbidity and mortality that necessitate prompt recognition and management.

**Case presentation:**

We present a case of a 46-year-old gentleman from Bangladesh who presented to the emergency department with fever, chills, and right upper quadrant abdominal discomfort. Evaluation revealed elevated inflammatory markers with high blood glucose and a subdiaphragmatic lucency on a plain chest radiograph. The suspected underlying visceral infection was confirmed by abdominal ultrasonography and computed tomography which demonstrated an emphysematous abscess of 8 cm in diameter in the right liver lobe.

Because of clinical instability, the patient was admitted to the medical intensive care unit (MICU) where he received appropriate supportive management with antimicrobials and percutaneous drainage of the abscess. Cultures collected from blood, the abscess, and urine grew a sensitive strain of Klebsiella pneumoniae. During his stay in the MICU, he complained of dyspnea. A CT pulmonary angiography was suggestive of septic emboli. A few days later, the patient started to complain of left gluteal pain and an US revealed a deep left gluteal abscess which required drainage. Cultures of the pus grew the same sensitive strain of Klebsiella pneumoniae. After receiving 6 weeks of parenteral antimicrobial therapy a repeated US revealed complete resolution of the abscess in the liver. Outpatient follow up showed favorable recovery.

**Conclusion:**

Gas-forming pyogenic liver abscess (GFPLA) is a rare manifestation of pyogenic liver abscess that usually occurs in patients with poorly controlled DM. Despite appropriate evaluation, morbidity remains high therefore timely recognition and anticipation of complications is important.

## Background

Pyogenic liver abscess (PLA) is an infectious disease that mainly affects adult patients, particularly those with underlying gastrointestinal diseases such as diverticular, gallbladder or biliary diseases [1]. The epidemiology of the disease varies with geographical location, patient demography and underlying comorbidities. The incidence of PLA is estimated to be around 2.3 cases per 100,000 populations with a male to female ratio of 2.3–1.5 [1], [2], [3]. The hallmark of the disease is characterized by the presence of a pyogenic space-occupying-lesion in the hepatic tissue which can be classified according to its size, causative microorganism(s), or presence of gas. The main presenting symptoms are fever, fatigue, nausea and vomiting [4], [5]. Since the infection is associated with significant morbidity and mortality, prompt recognition and evaluation is warranted.

Gas-forming pyogenic liver abscess (GFPLA) is a rare subgroup of PLA which was first recognized by Smith in 1944 who described the presence of gas within a pyogenic abscess [6]. When compared to PLA, GFPLA is less frequent but is associated with significantly higher morbidity and mortality [7], [8]. Reported complications include septic shock, bacteremia, rupture, endophthalmitis, acute respiratory and renal failure, recurrent liver abscess and other metastatic infections [9]. Morbidity and mortality remain high despite appropriate evaluation and management, therefore timely recognition and anticipation of complications is lifesaving [4], [9]. Therapeutic options include broad-spectrum antibiotics, abdominal US or CT to confirm the diagnosis, aspiration of the contents of the abscess for culture and catheter drainage when necessary [10]. Repeated imaging with abdominal US or CT is indicated if sepsis persists after 1 week of appropriate therapy, while surgical intervention should be considered in patients who failed drainage [10]. After resolution of the infection, patients are recommended to undergo colonoscopy in view of the association of colorectal cancer in patients with *Klebsiella penumoniae* PLA [11].

In the case presented below, a patient with GFPLA complicated by septic pulmonary emboli and muscle abscess was managed to safe outcome with current available evidence in the literature.

## Case presentation

**Fig. 1:**
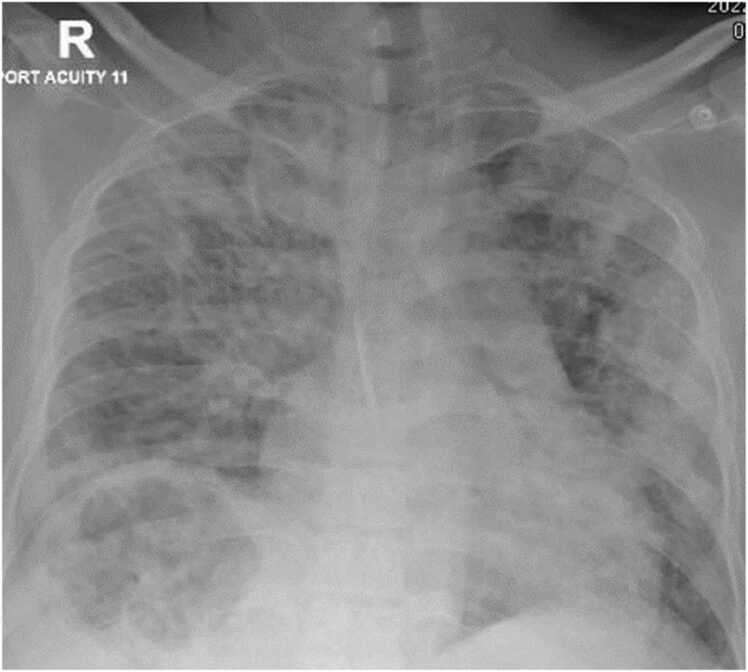
Chest X ray demonstrates bilateral airspace opacities and well-defined rounded lucency in the liver.

**Fig. 2:**
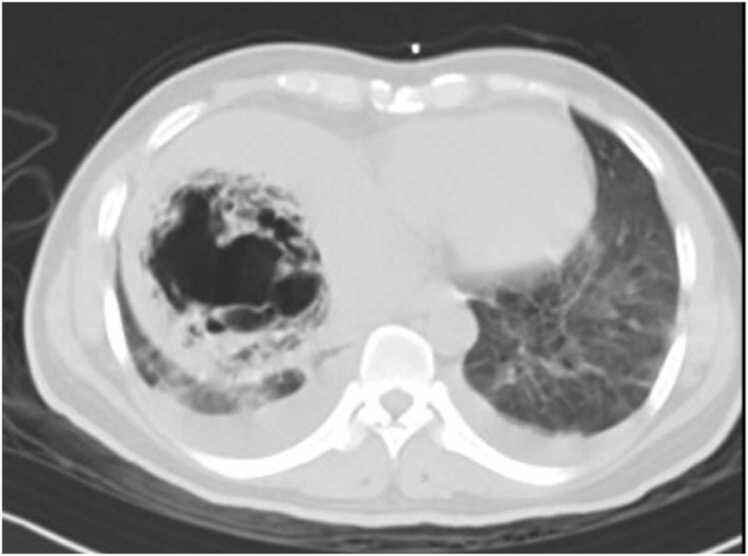
Abdominal CT demonstrates an abscess 8 cm in diameter in the right liver lobe and air tracking along the hepatic vein.

A 46-year-old gentleman from Bangladesh with no past medical history presented to the emergency department with a 3-day history of fever, chills and progressively worsening right upper quadrant abdominal discomfort. Upon initial assessment, he was febrile with a temperature of at 38.7 °C, blood pressure was 139/75, he was tachycardic and tachypneic with a heart rate of 155 beats per minute and respiratory rate of 33 breaths per minute, and his peripheral oxygen saturation was 94 % on room air. Physical examination revealed right upper quadrant tenderness without evidence of peritoneal irritation while heart sounds were normal with no audible murmurs and a chest examination was significant for reduced air entry to bases. Laboratory tests showed markedly elevated inflammatory markers with white blood cell count of 20.7 /all, C-reactive protein level of 315.8 mg/L and procalcitonin 95.10 ng/mL while HIV was excluded with a negative serology. Liver function tests were deranged with aspartate transaminase (AST) level of 142 U/L, alanine aminotransferase (ALT) level of 210 U/L and alkaline phosphatase (ALP) level of 486 U/L. In addition, blood glucose level was high at 410.4 mg/dL, glycated hemoglobin (HbA1c) level was 8.8 % but urine was negative for ketones and arterial blood gases showed no acidosis with a pH of 7.42. Radiological evaluation with a chest x-ray revealed bilateral patchy air space opacities and a well-defined rounded heterogenous hepatic lucency (Fig. 1). Hepatic pathology was confirmed with an abdominal ultrasonography (US) and a computed tomography (CT) scan that demonstrated a significant right lobe liver abscess 8 cm in diameter with a gaseous core replacing normal hepatic parenchyma and an air fluid levels as well as focal emphysematous air tracking into adjacent hepatic veins (Fig. 2).

Because of his critical condition, the patient was admitted to the medical intensive care unit (MICU) where he was started on empiric antibiotic therapy with high dose meropenem, and an insulin infusion for hyperglycemia followed by an urgent percutaneous catheter drainage of the abscess. A culture from the drained purulent fluid was significant for profuse growth of Klebsiella pneumoniae which was susceptible to standard antimicrobials therapy (Table 1) while cytology did not reveal any malignant pathology. Furthermore, blood and urine cultures collected at the time of presentation, grew the same sensitive strain of Klebsiella pneumoniae. Based on the antimicrobial susceptibility tests, antibiotics were adjusted to ceftriaxone 2 g/day in addition to adjuvant metronidazole therapy.

**Table 1:** *Klebsiella pneumoniae* sensitivities.

Drug	Antimicrobial sensitivity
Amoxicillin/clavulanate Ampicillin Cefuroxime Gentamicin Trimethoprim-Sulfamethoxazole	Susceptible Resistant Susceptible Susceptible Susceptible

**Fig. 3:**
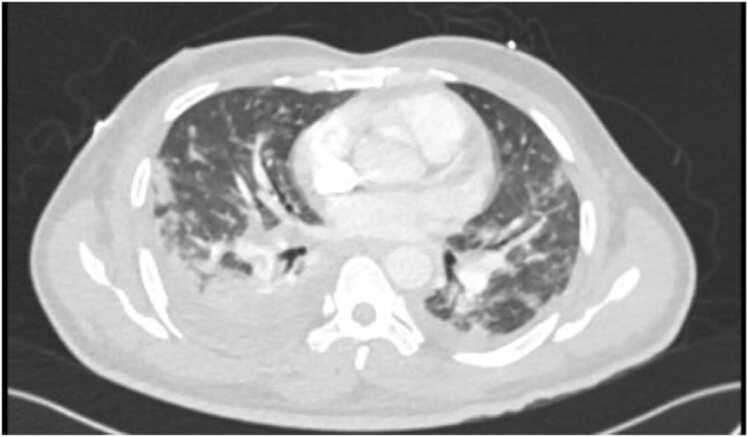
CT pulmonary angiography demonstrates bilateral pleural effusions and filling defects in the right lower lobe pulmonary artery division as well as scattered pulmonary nodules and irregular airspace infiltrates of varying sizes.

During his stay in the MICU, the patient became acutely tachypneic and tachycardic. An urgent CT pulmonary angiography (CTPA) revealed bilateral pleural effusions and filling defects in the right lower lobe of the pulmonary artery denoting probable septic emboli (Fig. 3). The patient continued to receive appropriate antimicrobial therapy. Subsequently, he remained hemodynamically stable, with improving inflammatory markers while repeated cultures demonstrated no further bacterial growth and he was eventually transferred out of the MICU to the general medical ward (GMW).

**Fig. 4:**
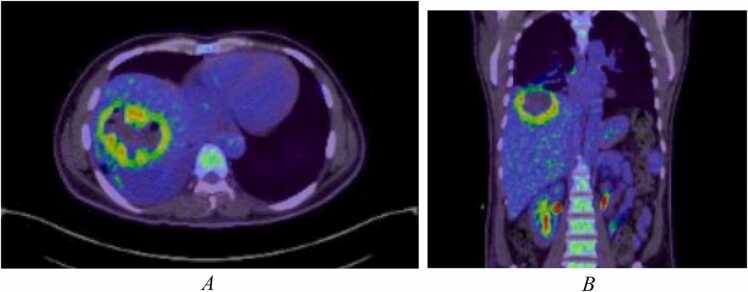
A and B PET CT FDG uptake scan demonstrates a large lesion in the right liver lobe segment VII, VIII measuring 7 cm by 7.5 cm with increased FDG uptake along its peripheral thick wall with air locules seen within the lesion.

**Fig. 5:**
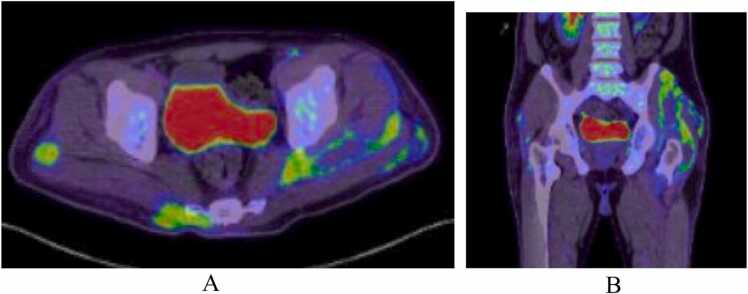
A and B PET CT FDG uptake scan demonstrates an ill-defined collection with peripheral increased FDG uptake and central necrosis, involving gluteus maximus, gluteus medius, and minimus, measuring 15 cm by 8 cm by 15 cm in maximal dimensions. There is also a focal area of fat stranding in the subcutaneous fat of the right gluteal region with peripheral FDG uptake measuring about 5 cm by 3.3 cm by 10 cm in maximal dimensions.

A few days later, the patient started to complain of left gluteal pain and developed a fever of 38.0 °C. An US revealed a deep left gluteal abscess measuring 42.6 mm by 4 mm with associate subcutaneous edema which required US guided drainage. Cultures of the purulent aspirated material grew the same sensitive strain of Klebsiella pneumoniae. A whole body fluorodeoxyglucose (FDG) positron emission tomography (PET) CT redemonstrated the liver abscess, pulmonary infarcts and necrotizing myositis in the left gluteus maximus, gluteus medius, and minimus, as well as a focal area of fat stranding in the subcutaneous fat of the right gluteal region suggesting superficial cellulitis and early abscess formation. There were no other sites with pathologic FDG uptake (Figs. 4 and 5). After drainage, his fever subsided and he remained vitally stable for several days at which time he was discharged from the hospital with outpatient antimicrobial therapy (OPAT) of ceftriaxone 2 g/day and general infectious diseases clinic follow up.

After receiving 4 weeks of parenteral ceftriaxone 2 g/day the abdominal US was repeated only to find that the abscess had barely decreased in size. He was therefore electively admitted to the hospital again where he underwent percutaneous aspiration of the liver abscess for further bacteriologic data. The aspirated material grew the same sensitive strain of Klebsiella pneumoniae. A transthoracic echocardiography (TTE) did not reveal any vegetations on the valves and a fundoscopic examination ruled out endophthalmitis. Ceftriaxone was changed to parenteral ertapenem 1 g/day for 2 weeks and a subsequent abdominal US revealed complete resolution of the previously seen abscess in the liver. The patient is currently doing well with regular follow ups with general medicine and is schedule to undergo a colonoscopy in a few weeks’ time to rule out the presence of colorectal cancer.

## Discussion

Pyogenic liver abscesses (PLA) are the most common type of visceral abscesses [1]. The liver is particularly susceptible to abscess formation since it receives blood from both; the portal and systemic circulations [12]. Bacterial organisms can travel to the liver through the portal vein which drains intrabdominal structures, particularly luminal gastrointestinal tract (GIT) tissues. Therefore, GIT diseases such as diverticular or biliary diseases as well as intrabdominal infections such as appendicitis and local collections pose a significant risk for developing liver abscesses [12]. Similarly, patients with DM, chronic renal or liver diseases as well as immune suppression and organ transplantations are at higher risk of acquiring liver abscesses [12]. When compared to PLA, GFPLA, a rare subgroup of PLA characterized by the presence of gas in the abscess, is potentially life-threatening with mortality rates ranging from 25.7 % to 37.1 % [9]. The presence of gas has led to the hypothesis that the formation of the emphysematous abscess involves the fermentation of mixed acids by bacterial enzymes, which, in turn, generate gaseous substances as a byproduct [7], [4]. These gaseous substances accumulate as an emphysematous collection within the compact hepatic parenchyma [4].

GFPLA was first described by Smith [6] in 1944, in an article that highlighted the high mortality rate associated with GFPLA in the elderly population. The average age of patients presenting with GFPLA was reported to be at around 56 years of age with a male predominance and higher mortality rates associated with older age [2], [3]. Some studies reported a strong association between GFPLA and DM ranging between 74 % and 95 %, however, data was conflicting regarding the association of GFPLA with poorly controlled DM [2], [7], [8], [3]. It is worth highlighting that some studies demonstrated the absence of abdominal pain on presentation, rather, fever and general malaise were the most common chief complaints [4], [5].

In terms of pathogens, the most commonly isolated organisms from pus cultures were *Klebsiella pneumoninae* and *Escherichia coli,* while *Pseudomonas aeruginosa* and gram-positive bacteria were less frequently isolated [13], [3], [14]. Blood cultures were positive in 87–100 % of patients with GFPLA and the most reliable radiological investigations were abdominal US or abdominal CT scans with a reported detection rate of 100 % while plain radiographs had a sensitivity rate of 25.7 % [2], [7], [13], [3]. In the patient presented above, a plain radiograph was suggestive of GFPLA while abdominal US and CT where confirmative.

Another point of interest is the high rate of complications associated with GFPLA which is reported to be as high as 90 % in some studies [2], [3], [15], [16]. These complications include bacteremia, septic shock, acute kidney injury (AKI), respiratory failure, abscess rupture, recurrent abscesses as well as metastatic infections to the eyes, lungs and muscle tissue [9], [17]. Of note, because of the proximity of the hepatic tissues to the thorax, pulmonary complications were common; reported to be as high as 60 % with preponderance of septic pulmonary emboli [4]. In contrast, metastatic infections to the muscle were less frequent [17]. Mortality rates among patients with GFPLA are also high; reported at around 30–40 % which is more than three folds the mortality rate among patients with typical PLA [9]. Predictors of mortality include elevated creatinine level and elevated blood sugar level [9]. Radiologically, pneumoperitoneum and alveolar gas pattern were the most significant mortality predictors [9]. On CT the most significant predictors were globular configuration of the abscess, shaggy abscess margins and total gas volume within the abscess [9].

Case reports of patients with *Klebsiella pneumoninae* endocarditis described similar presentations to the patient presented above [18], [19]. However, *Klebsiella pneumoninae* endocarditis is rare; it accounts for less than 5 % of all cases of endocarditis [18], [19]. Furthermore, a systematic review looking into the clinical characteristics and causes of septic pulmonary emboli found that out of 107 patients who were evaluated with echocardiography for suspected endocarditis, transesophageal echocardiography (TEE) revealed vegetations that were not initially visible on a TTE in only 6 cases [20]. Considering the rarity of the disease, a normal TTE, a known primary infective focus (a liver abscess) and a lack of supportive presenting symptoms of endocarditis, a TEE was deemed unnecessary.

Treatment of choice entails the combination of radiologically assisted percutaneous drainage or aspiration in addition to effective antimicrobial therapy tailored to identified pathogen(s) [21], [10], [22]. The combined approach of drainage and antibiotics administration is a safe and effective approach even in critically ill patients [23], [24], [25]. When percutaneous drainage fails, surgical drainage can be considered [21]. Antimicrobial therapy alone is controversial with failure rates reported to be as high as 42 % [1]. The choice of empiric antimicrobial therapy should cover *Enterobacteria* and should be tailored according to local antibiograms. Third generation cephalosporins in addition to anaerobic cover, such as metronidazole, can be standard cover while piperacillin-tazobactam or carbapenems such as meropenem can be also be considered. Following identifications of causative organism(s), antimicrobial therapy must be adjusted according to available susceptibilities taking into consideration potential none-isolated pathogens such as anaerobic bacteria. Risk factors for treatment failure include multiple abscesses, large abscesses (> 5 cm), presence of necrotic tissue, blockage of drainage catheter and technical difficulties while performing drainage [26].

## Conclusion

Gas-forming pyogenic liver abscess (GFPLA) is a rare manifestation of the pyogenic liver abscess. The condition is often associated with diabetes mellitus while enterobacteria such as *Klebsiella pneumoninae* and *Escherichia coli* are the main isolated pathogens. Treatment should be tailored to identified pathogens and patients usually require a prolonged antimicrobial course which depends on clinical and radiological progress, while augmented aspiration of sizable lesions is essential to reduce the burden of the disease. Despite initial appropriate assessment, significant morbidity and mortality remain high therefore, prompt recognition, detailed evaluation and management including anticipations of distant complications are essential precautionary measures.

## CRediT authorship contribution statement

AHA wrote the initial draft of the research and edited subsequent drafts. AAA reviewed and edited subsequent drafts. NA reviewed and edited subsequent drafts. HA reviewed and edited subsequent drafts. MA reviewed and edited subsequent drafts. All authors read and approved the final manuscript.

## Ethical approval

Approved by Hamad Medical Corporation Medical Research Centre proposal ID MRC-04-22-137.

## Consent

Written informed consent was obtained from the patient for publication of this case report and accompanying images. A copy of the written consent is available for review by the Editor-in-Chief of this journal on request.

## Declarations

None.

## Ethics approval and consent to participate

Not applicable.

## Funding

This research has not received any funding.

## Declaration of Competing Interest

The authors declare that they have no known competing financial interests or personal relationships that could have appeared to influence the work reported in this paper.
